# Adult neurogenesis and its anatomical context in the hippocampus of three mole-rat species

**DOI:** 10.3389/fnana.2014.00039

**Published:** 2014-05-20

**Authors:** Irmgard Amrein, Anton S. Becker, Stefanie Engler, Shih-hui Huang, Julian Müller, Lutz Slomianka, Maria K. Oosthuizen

**Affiliations:** ^1^Division of Functional Neuroanatomy, Institute of Anatomy, University of ZürichZürich, Switzerland; ^2^Department of Zoology and Entomology, University of PretoriaPretoria, South Africa

**Keywords:** Bathyergidae, calcium-binding proteins, Cape mole-rat, comparative neuroanatomy, highveld mole-rat, naked mole-rat, neurogenesis, stereology

## Abstract

African mole-rats (family Bathyergidae) are small to medium sized, long-lived, and strictly subterranean rodents that became valuable animal models as a result of their longevity and diversity in social organization. The formation and integration of new hippocampal neurons in adult mammals (adult hippocampal neurogenesis, AHN) correlates negatively with age and positively with habitat complexity. Here we present quantitative data on AHN in wild-derived mole-rats of 1 year and older, and briefly describe its anatomical context including markers of neuronal function (calbindin and parvalbumin). Solitary Cape mole-rats (*Georychus capensis*), social highveld mole-rats (*Cryptomys hottentotus pretoriae)*, and eusocial naked mole-rats (*Heterocephalus glaber*) were assessed. Compared to other rodents, the hippocampal formation in mole-rats is small, but shows a distinct cytoarchitecture in the dentate gyrus and CA1. Distributions of the calcium-binding proteins differ from those seen in rodents; e.g., calbindin in CA3 of naked mole-rats distributes similar to the pattern seen in early primate development, and calbindin staining extends into the stratum lacunosum-moleculare of Cape mole-rats. Proliferating cells and young neurons are found in low numbers in the hippocampus of all three mole-rat species. Resident granule cell numbers are low as well. Proliferating cells expressed as a percentage of resident granule cells are in the range of other rodents, while the percentage of young neurons is lower than that observed in surface dwelling rodents. Between mole-rat species, we observed no difference in the percentage of proliferating cells. The percentages of young neurons are high in social highveld and naked mole-rats, and low in solitary Cape mole-rats. The findings support that proliferation is regulated independently of average life expectancy and habitat. Instead, neuronal differentiation reflects species-specific demands, which appear lower in subterranean rodents.

## Introduction

African mole-rats are members of the family Bathyergidae within the Order Rodentia. Despite their common name, they are closer related to porcupines than to rats or moles (Skinner and Chimimba, 2005). All members of this family are endemic to sub-Saharan Africa, are strictly subterranean and feed on tubers and bulbous plants. Continuous digging in shallow tunnels supply the animals with food, the deeper burrows contain nests, toilet areas and, depending on the species, food stores and deep blind tunnels for protection and thermoregulation (Jarvis and Bennett, 1991). Burrows are usually sealed, and animals rarely if ever leave their tunnel system (Jarvis and Bennett, 1991). The subterranean habitat with its reduced sensory stimuli has been linked to the overall small brain size in mole-rats (Mace et al., 1981). The reduction of sensory areas in the brain has been studied in detail in the visual system. Most mole-rats still possess morphologically normal eyes, but they are microphthalmic (Hetling et al., 2005) and visual areas in the brain are reduced (Němec et al., 2008).

Unique for a rodent family, bathyergids show a wide spectrum of social organization, ranging from solitary to eusocial species (Bennett and Faulkes, 2000). The eusocial naked mole-rat in particular has become a popular animal model because of a resistance to develop tumors (Liang et al., 2010), successful ageing despite high oxidative cellular stress (Andziak et al., 2006) and adaptations to a hypoxic environment (Jarvis and Bennett, 1991). On a cellular level, the latter is achieved by, e.g., an attenuated calcium response in hippocampal pyramidal cells (Peterson et al., 2012). All mole-rat species are also known for their long life expectancies. Systematic field data are not available, but in captivity, naked mole-rats have been shown to reach an age of 32 years (Edrey et al., 2012), and also other mole-rat species can reach an age of 9–16 years in captivity (Dammann et al., 2011; Tacutu et al., 2013).

Adult hippocampal neurogenesis (AHN) is a well described trait in many mammals including humans (Kempermann, 2011). Of particular interest is the postnatal decline in proliferation of stem/progenitor cells that is dependent on absolute age, irrespective of lifespan (Amrein et al., 2011). The number of proliferating cells, which corresponds to roughly 5% of the resident granule cells around birth, declines exponentially within the first postnatal months. There are indications that proliferation in the mammalian dentate gyrus becomes relatively stable between the ages of 1 and 2 years, a time by which laboratory rodents have reached their average or maximum live span. To model human AHN, a small mammal that lives considerably longer than common laboratory rodents but that has an AHN showing similar regulatory mechanisms would be of great interest. Some sharing of regulatory mechanisms is suggested by a recent study of AHN in naked mole-rats (Peragine et al., 2014). It was shown that subordinate, non-breeding naked mole-rats have more young neurons than the dominant, breeding female. Although this observation was interpreted in terms of dominance relations, it also agrees with substantial evidence from wild and laboratory rodents that shows a transient or lasting negative impact of reproduction and maternal behavior on AHN (Galea and McEwen, 1999; Leuner et al., 2007; Pawluski and Galea, 2007; Cavegn et al., 2013).

The first aim of this study is to investigate AHN in mole-rats and evaluate its extent with respect to other mammalian species. Second, we proposed that the number of surviving young neurons is positively correlated with habitat complexity (Cavegn et al., 2013). We therefore hypothesize that the simple habitat associated with a subterranean life style would be accompanied by relatively low numbers of young differentiating neurons. Lastly, we are interested in a relationship between social complexity and AHN in rodents, a question that has been addressed experimentally *within* species of rodents (Gheusi et al., 2009) and birds (Barnea and Pravosudov, 2011). We therefore investigated the solitary Cape mole-rat (*Georychus capensis*), the social highveld mole-rat (*Cryptomys hottentotus pretoriae)* that occurs in small colonies with one breeding pair and up to 12 subordinates, and the eusocial naked mole-rat (*Heterocephalus glaber*) that exhibits the highest level of social organization, which includes hierarchical classification, cooperative breeding, division of labor, and overlapping generations within colonies with up to 100 individuals (Jarvis, 1981). To provide an anatomical context for AHN, we describe hippocampal cytoarchitecture including the expression patterns of the calcium-binding proteins calbindin and parvalbumin. Quantitative data of dentate gyrus granule cells, proliferating cells and young differentiating neurons in the subgranular layer of the three mole-rat species are discussed with respect to age-related changes and species-specific differences.

## Materials and methods

### Animals

Highveld mole-rats (*Cryptomys hottentotus pretoriae*) where trapped in Centurion, Gauteng, South Africa, and Cape mole-rats (*Georychus capensis*) were trapped near Darling, Western Cape, South Africa. Naked mole-rats (*Heterocephalus glaber*) were obtained from a colony of wild-derived animals maintained at the University of Nairobi, Kenya. Trapping and animal treatment were in accordance with ethical principles and guidelines for scientific animal experiments of the respective Universities. None of the females were pregnant or lactating, and no queens (the only reproductively active females) from the colonies of highveld and naked mole-rats were included in the study. Animals were weighed, sexed and, after deep anesthesia using Pentobarbital (50 mg/kg body weight), perfused transcardiacally with phosphate buffered saline (PBS), followed by sodium sulfide solution and fixed with 4% paraformaldehyde (PFA). Brains were removed and post-fixed overnight. Right hemispheres where cryoprotected in 30% sucrose and processed for immunohistochemistry. Left hemispheres were stored in PFA until methacrylate embedding. Femurs or teeth where collected for age determination. Tissue processing followed protocols described in detail previously (Cavegn et al., 2013).

### Histology

Sagittal, 40 μm sections of the right hemispheres were collected in series and stored in a cryoprotective solution until processing. Optimal concentrations for each antibody and species were determined in pilot studies to produce a strong signal and minimal background staining. For immunohistochemistry, free floating sections were stained for proliferating cells using polyclonal rabbit NCL-Ki67p, Novocastra, for naked (1:2500), Cape (1:3000), and highveld (1:5000) mole-rats and monoclonal mouse Ki67 (BD, 1:600) in highveld mole-rats. For visualization of young differentiating neurons, doublecortin (DCX, polyclonal goat IgG sc-8066, Santa Cruz Biotechnology, all species 1:1000) and monoclonal mouse anti- polysialylated neuronal cell adhesion molecule IgG (PSA-NCAM), Chemikon (1:6000 for highveld, 1:2500 for Cape and 1:2000 for naked mole-rats) were used. Calcium-binding proteins were visualized with IgGs against parvalbumin (monoclonal mouse IgG, P-3171, Sigma Aldrich, 1:5000 for naked and highveld, 1:10,000 for Cape mole-rats) and calbindin (polyclonal rabbit IgG, CB-38a, Swant, all species 1:20,000). Briefly, sections were washed with Tris-Triton [Tris-buffered saline (TBS), pH 7.4, with 0.05% Triton] and incubated in citrate buffer (Target Retrieval Solution, Dako; 1:10) for epitope retrieval either by microwave (DCX, PSA-NCAM, calbindin) or heat-treatment in save-lock tubes for 45 min at 90°C (Ki67). Parvalbumin staining did not require epitope retrieval. Incubation in Tris-Triton containing 2% normal serum, 0.2% Triton and primary antibodies was done overnight. Incubation in secondary antibodies (all 1:300, Vectastain; diluent as for primary antibody plus 0.1% bovine serum albumin) was followed by incubation with ABC solution (Vectastain). Finally 3,3′-diaminobenzidine (DAB) stained sections were mounted, dehydrated and cover-slipped.

For the estimation of granule cell numbers and general cytoarchitectonic evaluation, left hemispheres were washed with PBS and dehydrated in graded ethanols. After incubation in 1:1 ethanol and 2-Hydroxyethylmethacrylat (HEM, Technovit 7100, Kulzer) solution, hemispheres were infiltrated in three consecutive changes of HEM solutions for several days until embedding. 20 μm horizontal sections were cut and stained with Giemsa-solution (Merck) diluted 1:10 with 67 mmol KH_2_PO_4_ for 40 min at room temperature, differentiated in KH_2_PO_4_ for 90 s, dehydrated and cover-slipped. In naked and Cape mole-rats, parallel series of HEM-embedded sections were Timm-stained in a solution of 120 ml gum arabic (50% weight/volume in distilled water), 20 ml citrate buffer (pH 5), 60 ml of 0.5% hydroquinone, and 1 ml of 17% silver nitrate. Incubation at 37°C was followed by rinsing in tap water, fixation for 1 min with 1% sodium thiosulfate. Sections were counterstained with toluidine blue, dehydrated, cleared and cover slipped.

### Quantification

**Figure 1:**
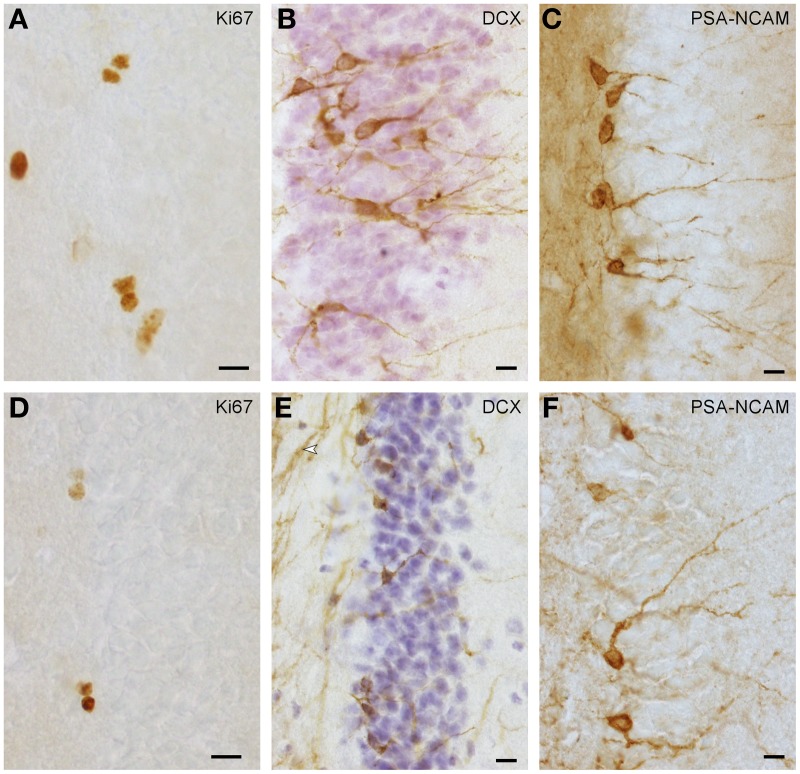
**Ki67+ proliferating cells (A,D) and DCX+ (B,E) or PSA-NCAM+ (C,F) young neurons in naked mole-rats (A–C) and highveld mole-rats (D–F)**. Note the staining of presumably myelinated fibers in **(E** arrow**)**, which in some sections did not allow the identification of DCX+ cells in highveld mole-rats. Background in PSA-NCAM stained sections of naked mole-rats was considerably higher than in highveld mole-rats obscuring weakly stained cells. We therefore choose DCX for the quantitation of young neurons in naked mole-rats and PSA-NCAM for highveld mole-rats and Cape mole-rats (not shown). **(A,E)** are composites of two focal planes. Scalebars: 10 μm.

Proliferating cells (Figures 1A,D) were quantified in every 6th section in highveld and Cape mole-rats and in every 5th section in naked mole-rats. On average, 16 (*SD* = 3) sections per animal were analyzed. Due to low cell numbers, quantification was exhaustive using a 100× oil immersion lens (NA = 1.3). Positively stained cells in the top focal plane were not considered to prevent over-counting. Total counts were multiplied with the inverse of the section sampling fraction. Quantification of young neurons was done as for proliferating cells, but in highveld mole-rats only every 12th section was used. PSA-NCAM staining (Figure 1F) provided the best signal in Cape and highveld mole-rats, whereas DCX staining (Figure 1E) did generate a fibrous background staining that could make it difficult to identify positive cells. In naked mole-rats DCX staining (Figure 1B) was used for quantification as it was more distinct than the PSA-NCAM (Figure 1C) and generated lower background staining. Data for total granule cells were assessed in the HEM embedded sections with the optical Fractionator (West et al., 1991) using Stereoinvestigator 10 (MBF Bioscience). Section sampling intervals were 12 for Cape and highveld mole-rats and 6 for naked mole-rats. For highveld mole-rats, a step size of 140 μm and dissector size of 12 × 12 × 10 μm was used, for Cape mole-rats a step size of 250 μm and dissector size of 15 × 15 × 10 μm, and for naked mole-rats a step size of 110 μm and dissector size of 10 × 10 × 10 μm.

### Age estimation

Relative age determination in Cape and naked mole-rats was based on the number of adhesion lines in the circumferential lamellae of the femur (Barker et al., 2003). Femurs were washed and decalcified in a rapid decalcifier (J. T. Baker, Histo Grade) for 24 h. A 3 mm segment was taken from the mid-diaphysis and infiltrated with HEM as described above. Alternate series of 20 μm sections were stained with hematoxylin-eosin and Schmorl's picrothionin. In the cross-sectioned femurs, adhesion lines were counted in the inner and outer lamellar bone tissue in both stains (Cavegn et al., 2013). In highveld mole-rats, age was based on tooth wear according the nine stages defined by Janse van Rensburg et al. (2004). All animals were classified with grade 5 or higher, indicating that all molars where fully erupted. In addition, body weight was compared with reported values (Janse van Rensburg et al., 2004). Bone growth, tooth wear and body weight data were analyzed with respect to the time of trapping and information of the seasonal breeding behavior in highveld mole-rats (Janse van Rensburg et al., 2002) and Cape mole-rats (Bennett and Jarvis, 1988). The naked mole-rats were trapped in the East Tsavo National Park, Kenya, in August 2008 and maintained at the University of Nairobi. At the time of investigation, the naked mole-rats had reached a minimum age of 3 years. For intra-species analysis, age scores were used for statistical analysis. For inter-species comparisons, animals were either classified into groups (around 1 year old, between 2 and 3 years old, more than 3 years old) or were assigned a tentative age in month based on their scores for the parameters described above.

### Statistics

Statistical analysis was performed with IBM SPSS (version 20). Within species, the effect of age on cell numbers was tested using a one-tailed Pearson correlation. Between species, a general linear model (GLM) was used with proliferation and young neurons expressed as a percentage of total granule cells (normalized cell numbers) as dependent variable, species as fixed factor and age scores as covariate. Using tentative age in months did not change the statistical outcome. Linear regression analysis was used to test for dependence of log transformed normalized proliferation and young neurons on log transformed age and species groups in mole-rats compared to data of a large number of rodents taken from previous studies (Amrein et al., 2011; Cavegn et al., 2013). *P*-values less than 0.05 were considered significant in all statistical comparisons. Graphs 3 and 4 were prepared with the R package ggplots2 (Wickham, 2009).

## Results

### Neurogenesis in mole-rats

#### Impact of lifestyle and life expectancy on AHN

**Figure 2:**
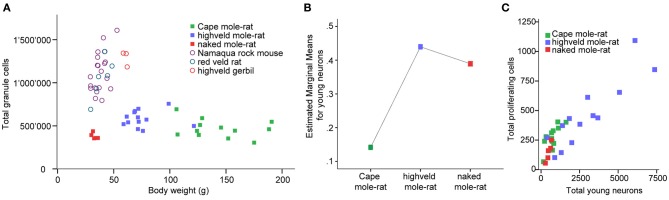
**(A)** Mole-rats have strikingly lower granule cell numbers than other African rodents [data taken from Cavegn et al. (2013)]. Also, granule cell numbers in mole-rats do not increase with body weight. Highveld mole-rats with intermediate body weight have more granule cells than the heaviest animals, the Cape mole-rats. **(B)** GLM plot of estimated marginal means of the percentage of young neurons relative to granule cells is high in the social highveld mole-rat and eusocial naked mole-rat, whereas values in solitary Cape mole-rat are low. The covariate age (mean tentative age of 26 months) was used in this model. **(C)** Scatterplot of estimated numbers of proliferating cells (Ki67) and young neurons (DCX or PSA-NCAM) in the mole-rats.

**Figure 3:**
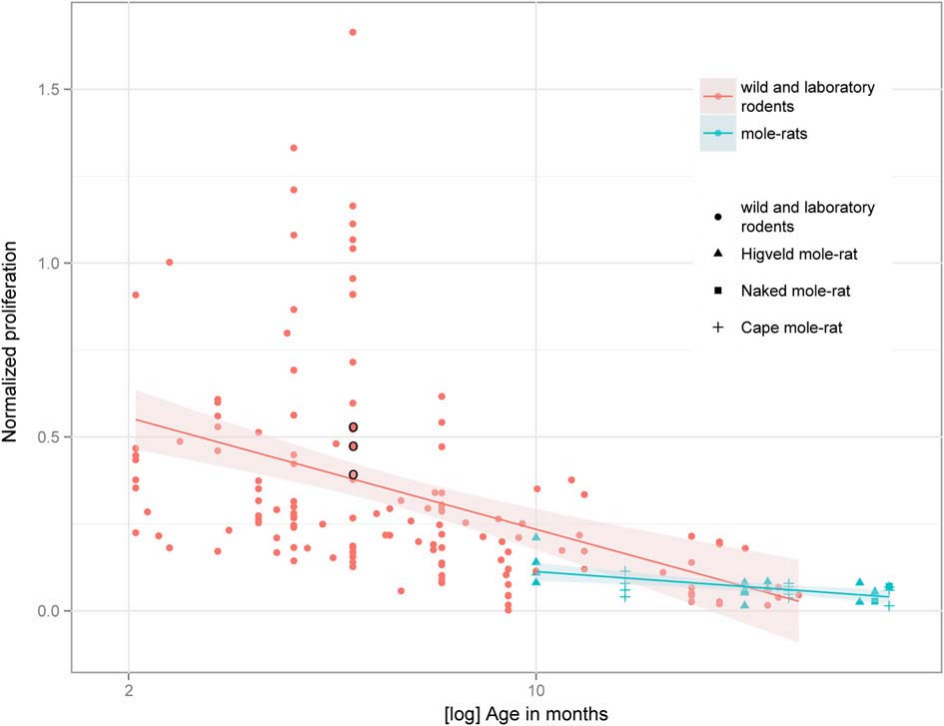
**Percentage of proliferating, Ki67+ cells relative to resident granule cells (normalized proliferation) relative to tentative age in mole-rats does not deviate from values collected in 13 species of surface dwelling wild and laboratory maintained rodents older than 2 months [data taken from Amrein et al. (2011) and Cavegn et al. (2013)]**. The only subterranean rodent other than mole-rats, the pine vole (*Microtus subterraneus*, indicated with black circles), also clusters with the other rodents. The age-dependent down-regulation is similar across surface-dwelling and subterranean rodents, and is also independent from average life expectancy.

**Figure 4:**
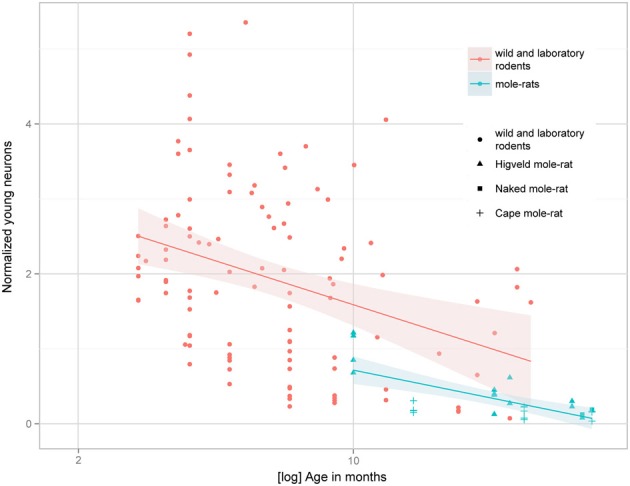
**Estimated numbers of young neurons (DCX+ or PSA-NCAM+) as a percentage of total granule cells (normalized young neurons) relative to tentative age are markedly lower in subterranean mole-rats compared with surface dwelling rodents**. Linear regression model of log-transformed data, *N* = 129, overall *F*-ratio 95.9, *p* < 0.001; β_age_ = −0.46, *p*_age_ < 0.001; β_habitat-group_ = −0.37, *p*_habitat-group_ < 0.001. Data for adult (older than 2 months) surface dwelling rodents of 13 different species are taken from Amrein et al. (2011) and Cavegn et al. (2013).

Despite the low absolute cell numbers, precision of the cell number estimates are good, and coefficients of error (CEs; Slomianka and West, 2005) do not exceed 13% (Table 1). In mole-rats aged around 1 year and older, hippocampal neurogenesis is low (Table 1, Figure 1). Absolute numbers of proliferating cells and young cells of the neuronal lineage are below of what has been reported for 12–24 months old Fischer 344 rats (Merrill et al., 2003; Rao et al., 2005, 2006). Low numbers of AHN-related cell types in mole-rats have to be seen in the context of low numbers of resident granule cells, which are also distinct from a sample of other South African rodents (Cavegn et al., 2013; Figure 2A). Normalized proliferating cells (proliferating cells as a percentage of resident granule cells) of subterranean mole-rats are surprisingly close to the data reported for surface dwelling, adult rodents (Amrein et al., 2011; Cavegn et al., 2013). Linear regression of log-transformed data show a decline in proliferation with estimated age in mole-rats that is similar to the decline of cell proliferation in 13 different species of rodents (Figure 3; *n* = 156, overall *F*-ratio 79.6, *p* < 0.001; β_age_ = −0.63, *p*_age_ < 0.001). Tested for the habitat groups (subterranean vs. surface dwelling), normalized proliferation does not differ between subterranean mole-rats and surface dwelling rodents (Figure 3; *n* = 156, overall *F*-ratio 79.6, *p* < 0.001; β_habitat−group_ = −0.11, *p*_habitat−group_ = 0.16). In contrast, the effect of habitat type on normalized numbers of young neurons separates the subterranean mole-rats from the surface dwelling rodents (Figure 4; log-transformed data, *n* = 129, overall *F*-ratio 95.9, *p* < 0.001; β_habitat−group_ = −0.37, *p*_habitat−group_ < 0.001). The decline in the number of normalized young neurons while aging (β_age_ = −0.46, *p*_age_ < 0.001) is as clear as that of proliferating cells.

**Table 1:** **Estimated cell numbers are given unilateral and rounded to the next 1000 (granule cells) or next 10 (proliferation and young neurons), values in parentheses indicate *SD***.

	**Body-weight in g**	**Brain-weight in g**	**Tentative age in months**	**Granule cells**	**CE granule cells**	**Young neurons**	**CE young neurons**	**Proliferating cells**	**CE proliferating cells**
**HIGHVELD MOLE-RAT**									
All animals (*n* = 13)	76.2 (17.0)	1.09 (0.09)	21.5 (9.4)	582,000 (94,000)	0.09 (0.02)	2300 (2100)	0.09 (0.03)	460 (280)	0.10 (0.04)
Females (*n* = 11)	70.0 (6.2)	1.06 (0.05)		574,000 (83,000)		3230 (2130)		470 (300)	
Males (*n* = 2)	110.7 (16.0)	1.25 (0.07)		627,000 (182,000)		1710 (1860)		440 (240)	
**CAPE MOLE-RATS**									
All animals (*n* = 12)	144.6 (29.3)	NA	25 (9.5)	468,000 (106,000)	0.11 (0.03)	750 (410)	0.06 (0.02)	270 (100)	0.12 (0.05)
Females (*n* = 10)	148.2 (31.0)	NA		466,000 (116,000)		710 (430)		280 (100)	
Males (*n* = 2)	126.1 (2.0)	NA		475,000 (49,000)		930 (270)		260 (130)	
**NAKED MOLE-RAT**									
All animals (*n* = 5)	33.3 (2.3)	0.4	40 (2.8)	380,000 (35,000)	0.08 (0.01)	500 (150)	0.10 (0.04)	150 (80)	0.13 (0.04)
Female (*n* = 1)	36.4	0.4		359,000		620		180	
Males (*n* = 4)	32.6 (1.7)	0.4		386,000 (38,000)		465 (160)		140 (80)	

Precision of the cell estimates is evaluated and presented as coefficients of error (CEs); NA: not assessed.

#### Age-depended regulation of neurogenesis in mole-rats

We observe an age-dependent decline in neurogenesis only in highveld mole-rats [cell proliferation: *r*_(11)_ = −0.69, *p* = 0.009; young neurons: *r*_(11)_ = −0.844, *p* < 0.001], but not in Cape mole-rats [cell proliferation: *r*_(10)_ = −0.288, *p* = 0.364; young neurons: *r*_(10)_ = −0.261, *p* = 0.413] or in naked mole-rats [cell proliferation: *r*_(3)_ = −0.13, *p* = 0.835; young neurons: *r*_(3)_ = −0.198, *p* = 0.75]. In none of the species does total granule cell number change with age. Our animal sample does not include young animals, in which the strongest age-dependent decline would be expected (Ben Abdallah et al., 2010), but confirms that neurogenesis becomes stable after the age of approximately 1 year. Even in the oldest animal in this sample, the naked mole-rats, we found proliferating cells that accounted for 0.04% (*SD* = 0.02) of all granule cells, which is in the range of adult primates (Gould et al., 1999).

#### Sociality and neurogenesis in mole-rats

Corrected for age, there is a main species effect on the number of normalized young neurons [*F*_(2, 26)_ = 7.64, *p* = 0.002]. The pairwise comparisons show that social highveld mole-rats and eusocial naked mole-rats score higher for normalized young neurons than solitary Cape mole-rats. The difference between social and solitary species is significant between highveld and Cape mole-rats (*p* = 0.001) but not between naked and Cape mole-rats (*p* = 0.054, Figure 2B). The two social species are not different from each other (*p* = 0.702). Normalized proliferating cells do not differ between the tree mole-rat species [*F*_(2, 26)_ = 0.544, *p* = 0.587]. Again corrected for age, the ratio of proliferating cells to young neurons is higher in Cape mole-rats (mean 0.449, *SD* = 0.054) than in highveld mole-rats (mean 0.223, *SD* = 0.055) and naked mole-rats (mean 0.204, *SD* = 0.102; see also Figure 2C). Pairwise comparisons indicate that Cape mole-rats differ in their ratio from highveld (*p* = 0.007) and naked mole-rats (*p* = 0.047), whereas the two social species cannot be distinguished from each other (*p* = 0.882).

### Hippocampal anatomy

#### Histoarchitecture

The hippocampal formation of mole-rats shows some features distinct from laboratory mice and rats. The observations are described for the naked mole-rats, and compared with the highveld and Cape mole-rats.

**Figure 5:**
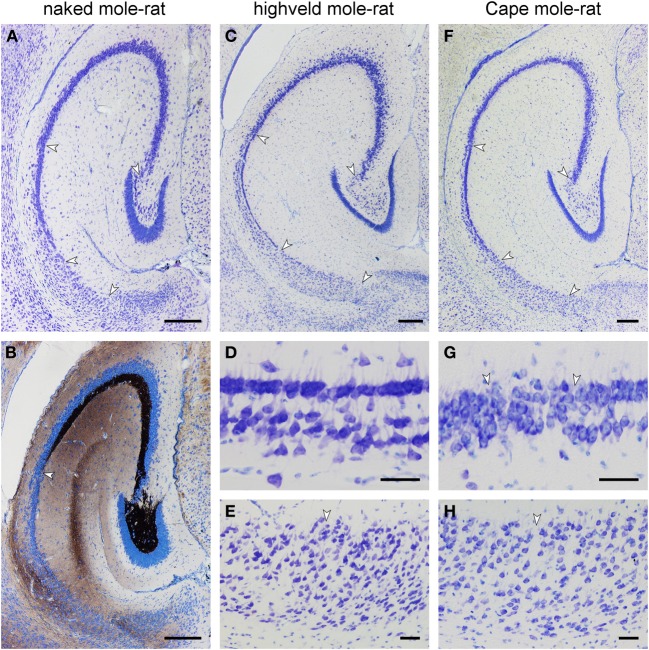
**Histoarchitecture of mole-rat mid-septotemporal hippocampi in Giemsa-stained horizontal sections (A) Naked mole-rat**. Arrows mark boundaries between hippocampal fields. Tiering of the CA1 pyramidal cell layer is less regular than in the other species. **(B)** Naked mole-rat, Timm-stained. The commissural/associational zone of the dentate molecular layer remains unstained. The cell poor part of the hilus is densely stained. The mossy fibers zone does not form a sharp end but thins out into scattered terminals. **(C)** Highveld mole-rat. Arrows mark boundaries between hippocampal fields. **(D)** Highveld mole-rat CA1. **(E)** Highveld mole-rat subiculum. **(F)** Cape mole-rat. Arrows mark boundaries between hippocampal fields. **(G)** Transition from Cape mole-rat CA3 to CA1. Arrows mark the boundaries of the narrow zone in which cells with the characteristics of CA1 and CA3 intermingle. **(H)** Cape mole rat subiculum. Arrow marks the boundary between the proximal and distal divisions of the subiculum. Scalebars: **(A–C,F)** = 250 μm; **(D,E,G,H)** = 50 μm.

In the naked mole-rat, the molecular layer of the dentate gyrus is very wide in comparison with the small dentate granule cell layer. Granule cells are separated by a thin hilar plexiform layer from the hilar polymorphic cells that form a narrow band below the granule cells (Figure 5A). The center of the hilus contains only very few cells. CA3 pyramidal cells do not insert between the blades of the granule cell layer, but join the suprapyramidal end of the hilar polymorphic cell band (Figure 5A). CA3 pyramidal cells occupy three-quarters of the entire hippocampal pyramidal cell layer. The short CA1 pyramidal cell layer has an irregular, loosely packed deep, and a denser superficial tier (Figure 5A). The subiculum is also short and only vaguely divided into proximal and distal parts. Timm-stained mossy fibers reveal a dense terminal field around the hilar polymorphic cells and also in the cell-sparse central part of the hilus (Figure 5B). Timm staining of the molecular layer is weak, restricted to the suprapyramidal area and confined to the outer layer (Figure 5B). A staining of the commissural/associational zone is not apparent. Timm-staining is unusually weak in the stratum oriens of proximal CA3. The mossy fiber staining gradually thins out into scattered terminals over a transitional zone in which pyramidal cells characteristic of CA1 and CA3 intermingle.

Highveld and Cape mole-rats share the well-developed molecular layer, small granule cell layer and extensive CA3 pyramidal layer with the naked mole-rats (Figures 5C,F). The hilar plexiform layer is in both species broader than in naked mole-rats—in particular below the suprapyramidal blade. Hilar cells of the polymorphic layer are not as dense as in naked mole-rats and scatter into the central part of the hilus. A continuity of hilar polymorphic and CA3 pyramidal cells is apparent as well (Figures 5C,F). CA1 is clearly separated into a deep and superficial tier, separated by a relatively cell-free zone (Figures 5D,G), along its entire septo-temporal and proximal-distal extent. The subiculum is wider than in naked mole-rats and proximal and distal parts are better defined. The proximal part contains a cell dense superficial and looser deep tier largely composed of pyramidal cells whereas cell morphologies are more diverse in the distal part (Figures 5E,H).

#### Parvalbumin

**Figure 6:**
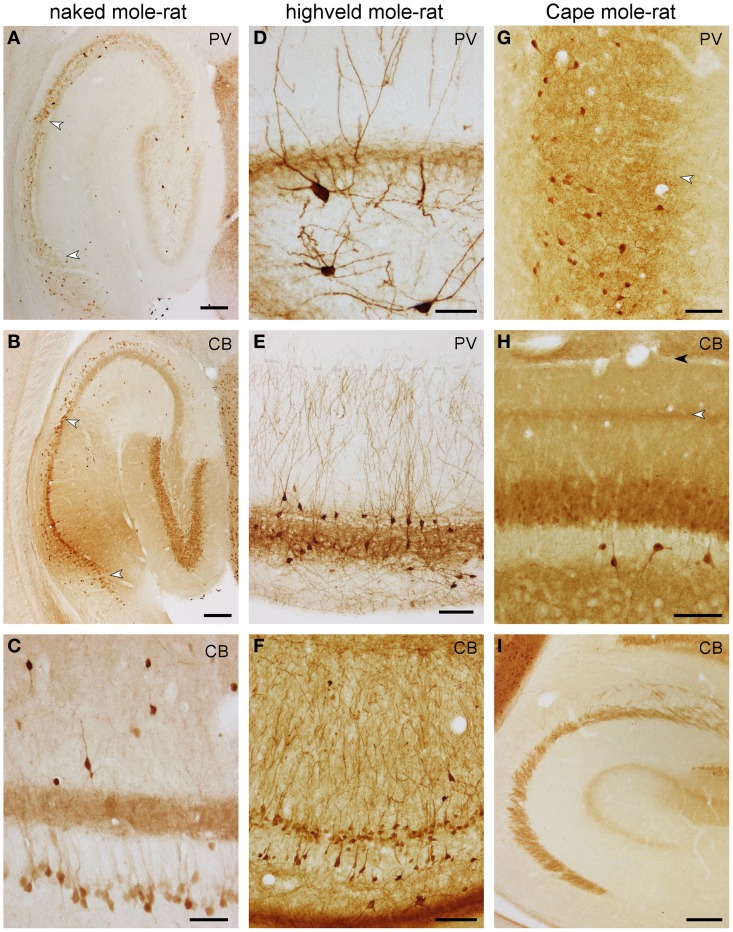
**Distribution of calbindin (CB) and parvalbumin (PV) in the hippocampus of mole-rats**. Parvalbumin **(A)** and calbindin **(B)** in the septal hippocampus of naked mole-rat. Arrows mark the CA1/CA3 and CA1/subiculum boundaries. Of particular note are deep calbindin+ pyramidal cells in the distal two-thirds of CA3. **(C)** Calbindin in the mid-septotemporal naked mole-rat CA3. Parvalbumin in the mid-septotemporal dentate gyrus **(D)** and CA1 **(E)** of the highveld mole-rat. Dendritic parvalbumin-immunoreactivity is unusually strong in this species. The shaded line in **(E)** marks the boundary between the CA1 stratum oriens and lacunosum-moleculare. **(F)** Calbindin in the mid-septotemporal highveld mole-rat CA1. **(G)** Parvalbumin in the mid-septotemporal Cape mole-rat subiculum. The arrow marks the border between the proximal and distal parts of the subiculum. Calbindin in the mid-septotemporal dentate gyrus **(H)** and CA3 **(I)** of Cape mole-rats. The light arrow in **(H)** marks the calbindin+ band of the dentate medial perforant path that extends into CA3; the dark arrow marks the boundary between the dentate molecular layer and CA1 stratum lacunosum moleculare. Scalebars: **(A,B,I)** = 200 μm; **(C,D)** = 50 μm; **(E–G)** 100 μm.

In naked mole-rats, terminal-like parvalbumin-staining is observed in the commissural-associational zone of the dentate molecular layer. The remainder of the molecular layer is not stained (Figure 6A). Faint scattered granules are seen in the dentate granule cell layer. Few bipolar or pyramidal and, rarely, multipolar parvalbumin+ cells are observed in the hilar plexiform and polymorphic cell layers septally. Temporally, most hilar polymorphic cells stain, albeit weaker, for parvalbumin. In CA3, few parvalbumin+ cells are associated with the pyramidal cell layer, their processes form an irregular network mostly associated with the cell layer, and its density is higher toward stratum oriens (Figure 6A). Dendrites of very few cells can be followed into the stratum radiatum. In CA1, parvalbumin+ cells are as few as in CA3, but almost exclusively confined to the deep tier of the pyramidal cell layer (Figure 6A). An irregular network of processes stains less intensely than in CA3 and is mainly located deep to the densely packed superficial CA1 pyramidal cells. Cells in the subiculum are both more frequent and staining darker than in other hippocampal fields. They are evenly distributed in the subicular cell and plexiform layers (Figure 6A).

Highveld mole-rats show considerably stronger parvalbumin staining than naked mole-rats. Many strongly parvalbumin+ cells are present within the deep granule cell layer and hilus (Figure 6D), which extend dendrites through the entire molecular layer. The molecular layer itself does not contain parvalbumin+ cells. Parvalbumin+ cells are found within or near CA3 and CA1 principal cell layer, as well as in the stratum oriens close to the alveus (Figure 6E). There, cells become more numerous in CA1. Stratum radiatum and lacunosum-moleculare rarely contain parvalbumin+ cells. Terminal-like staining is visible throughout the granule cell layer, CA3 and CA1 pyramidal layer but is, in contrast to naked mole-rats, not present in the commissural-associational zone of the dentate molecular layer (Figure 6D). In contrast to mouse control sections, the staining pattern of the mossy fiber layer is not different from stratum radiatum. Dendrites that originate from cells associated with the superficial pyramidal cell layer are oriented perpendicular to the pial surface and form a dense filigree in stratum radiatum of CA1 and CA3 (Figure 6E). Only very fine terminal branches cross into the stratum lacunosum-moleculare. Parvalbumin+ cells in the deep pyramidal cell layer and in the stratum oriens form a net-like dendritic pattern in the stratum oriens (Figure 6E). Darkly stained cells in the subiculum show a preference for the deep cell layer and are absent from the plexiform layer. Staining intensity for parvalbumin in Cape mole-rats is weaker, but the expression pattern is similar to that of highveld mole-rats. A distinct band of parvalbumin+ cells at the stratum oriens/alveus border is not present. Dendrites are stained much weaker than in the highveld mole-rat. The subiculum (Figure 6G) stains similar to naked and highveld mole-rats.

#### Calbindin

In naked mole-rats, even, light staining is seen in the molecular layer (Figure 6B). Dentate granule cells stain moderately. Very few calbindin+ cells (1–2 per section) can be found in the hilus. Neuropil staining in the hilar plexiform and polymorphic layers is similar to that of the molecular layer and is continuous with the CA3 mossy fiber layer (Figure 6B). A band of calbindin+ deep CA3 pyramidal cells is apparent in naked mole-rats only (Figures 6B,C). They are most prominent in distal CA3 septally and gradually extend into proximal CA3 temporally. Calbindin+ cells are scarce in the stratum oriens (~1 cell per section). Stratum radiatum contains few scattered darkly stained calbindin+ cells, which are bipolar or pyramidal with beaded dendrites that show no preferential orientation (Figure 6C). They are more common in the temporal hippocampus. In CA1, weakly stained, scattered deep pyramidal cells are separated from a continuous band of darker stained superficial pyramids (Figure 6B). Staining similar to the distal CA1 extends into the proximal subiculum, whereas few darkly stained cells are scattered in the distal subiculum.

The staining patterns of the highveld mole-rat molecular layer and principal cell layers (dentate gyrus and CA1; Figure 6F) are similar to that seen in naked mole-rats. Intensely stained, scattered bipolar and pyramidal calbindin+ cells in the stratum oriens and radiatum are more frequent than in naked mole-rats. Their dendrites are often perpendicular to the pial surface. Hippocampal calbindin staining is overall weakest in Cape mole-rats (Figure 6I), even though strong cortical neuronal and neuropil staining was observed. An inner and outer, moderately stained molecular layer is separated by a darker band that continues into the CA3 stratum lacunosum moleculare (Figures 6H,I). Dentate granule cell and CA1 pyramidal cell staining is weak. Strongly stained interneurons are only consistently found in the otherwise very light hilar plexiform layer (Figure 6H), in which they are more frequent than in the other two species, and in the stratum oriens of CA1. Similar to the naked mole-rat, strongly stained interneurons become more frequent in the temporal hippocampus, although they remain rare in layers other than CA1 stratum oriens. Subicular staining in highveld and Cape mole-rats resembles that of the naked mole-rat.

## Discussion

### A subterranean lifestyle associates with low numbers of young neurons

Of the three mole-rat species presented here, the naked mole-rat is the best researched and has attracted interest due to its resistance to cancer and longevity under low oxygen conditions (Liang et al., 2010; Kim et al., 2011). While the naked mole-rat shows extended habitat adaptations such as hairlessness and ectothermy (Alexander, 1991) other, less well studied mole-rat species have to deal with similar environmental conditions, and it is therefore of interest that the regulation of AHN in naked mole-rats does not deviate from the other mole-rats.

Normalized numbers of proliferating cells in mole-rats are not different from other rodents, which agrees with previous findings that age-related changes in proliferation follow a chronologically similar time course, independent of life expectancy and important life history events (Amrein et al., 2011). Compared to other rodents, normalized numbers of young neurons in mole-rats are lower, corroborating a habitat-dependent modulation of neuronal differentiation (Cavegn et al., 2013). The sealed tunnel systems with its limited sensory stimulation that mole-rats inhabit provide a constant and rather safe environment that does not require high behavioral flexibility. Sustaining many young neurons, which can mediate rapid behavioral adaptations to environmental challenges (Garthe et al., 2009; Amrein et al., 2011; Cavegn et al., 2013), may not be required in the mole-rats' relatively uniform habitat. It remains to be investigated whether low numbers of young neurons are due to a lack of survival-stimulating input during a critical time window (Shors et al., 2012) or an altered pace of maturation (Snyder et al., 2012). Of interest is that the reduction appears not only on the cellular level (reduced number of young neurons and granule cells) as shown here, but on a systems (small visual system, Hetling et al., 2005; Němec et al., 2008) and organ level (small brain size, Mace et al., 1981) as well. Brain size has been discussed in relation to habitat before. High climatic variability in temperature and precipitation is associated with larger brains in birds, while small-brained species are less tolerant to climatic variability and prefer rather stable habitats (Schuck-Paim et al., 2008). In mammals however, the stable environment of a subterranean lifestyle is not generally linked with small brains. Subterranean mole-rats (Bathyergidae) have small brains compared to closely related, surface dwelling species, but there are other examples, e.g., in the families of Octodontidae (degus and rock rats) and Talpidae (desmans and moles) in which relative brain size of subterranean species is similar to above ground living congeners (Mace et al., 1981). Thus, subterranean living conditions may not be the only explanation for the relatively small brains in mole-rats. In terms of neurogenesis, the only other subterranean mammals that have been investigated for AHN are the European mole (Bartkowska et al., 2010) and the pine vole (Amrein et al., 2004a). AHN in moles cannot be compared to the mole-rats as quantitative data was only provided for proliferating cells. Normalized cell proliferation in pine voles, as shown in Figure 3, does not deviate from other rodents, including mole-rats.

### Stable neurogenesis in long lived rodents

Hippocampal neurogenesis is relatively high at birth, but shows an exponential decline within the first postnatal months in both laboratory and wild mammals (Ben Abdallah et al., 2010; Amrein et al., 2011). Rats and mice aged 12 and 24 months show relatively low but stable neurogenesis (Heine et al., 2004; Rao et al., 2005; Kronenberg et al., 2006). The youngest highveld mole-rats included in this study were born in the previous breeding season which was ~10 months before trapping, the youngest Cape mole-rats were at least 14 months old. The sample of naked mole-rats consists of animals that were at least 3 years old. Since the highveld mole-rats were the only species that included animals less than 1 year old, it is not surprising that an age-dependent decline in AHN was seen only in this species. Neurogenesis in Cape and naked mole-rats is likely to have reached a low but stable level. Observing AHN in healthy, reproductively active rodents with a long life expectancy makes them an interesting translational animal model with the potential to extend the knowledge of AHN regulation in shorter lived laboratory mice and rats to the first decade of longer lived species.

### AHN and sociality

It is well known that experimental alterations of the social context impact AHN in several mammals (Gheusi et al., 2009; Lieberwirth and Wang, 2012) and birds (Barnea and Pravosudov, 2011). Starting points of these studies are however *social* animals that are experimentally maintained solitary, in pairs or in groups. There are few studies that compared solitary with social species. Fowler et al. (2005) found a higher density of proliferating cells in solitary meadow voles compared to the social prairie voles. Higher numbers of proliferating cells in the asocial yellow-necked wood mice than in social bank or pine voles have been reported as well (Amrein et al., 2004b). In contrast, Barker et al. (2005) found more proliferating cells in social Eastern gray squirrels than in asocial Yellow-pine chipmunks. Likewise, Snyder et al. (2009) found higher proliferation in laboratory rats compared to laboratory mice. Under natural conditions rats would life in mixed-gender groups, while mice organize in groups of one territorial male with several females. In the present study and when age is taken into account, hippocampal proliferation does not deviate between the solitary Cape mole-rat, social highveld mole-rat and eusocial naked mole-rat. In all three species, the number of proliferating cells corresponds to less than 0.1% of the resident granule cells. Thus, there appears to be no coherent relation between cell proliferation and sociality.

A different picture emerges for the number of young neurons. The higher proliferation in rats was accompanied by a higher number of young neurons when compared to mice (Snyder et al., 2009). We previously discussed the potential of sociality to shape neuronal differentiation on a phylogenetic scale based on high numbers of young neurons observed in highly social foxes and spiny mice (Amrein et al., 2011). In this diverse assortment of species, differences other than sociality will always be potential confounders. However, the social highveld mole-rats and eusocial naked mole-rats score higher for young neurons than the solitary Cape mole-rats, and the relation to proliferating cells also indicates that the social species show increased neuronal survival or an extended period of neuronal differentiation. In the case of mole-rats, animals are closely related and share very similar habitats. Although the species sample is still too small to draw firm conclusions, sociality is at least an incipient factor that may impact on AHN by way of regulating the number of young neurons. In line with this suggestion, an increase in AHN by housing socially isolated mice in an enriched environment is associated with the rescue of an impairment in long-term social memory (Monteiro et al., 2014).

### Hippocampal histoarchitecture and calcium-binding proteins

The dentate molecular layer in mole-rats is surprisingly large, its characteristics of laminar organization appears to deviate from the normal rodent pattern. A zinc-positive commissural-associational zone as seen in rats and mice (Haug, 1974; Slomianka and Geneser, 1997) is not evident in naked and Cape mole-rats, and also not in hedgehogs (West et al., 1984). The accentuated calbindin staining of the medial perforant path and its continuation into the CA3 stratum lacunosum-moleculare in Cape mole-rats has, to our knowledge, not been described in other species. All mole-rats show a quite small dentate gyrus granule cell layer that also contains fewer granule cells than in other rodents. The compact hilar polymorphic cell layer merges with CA3 pyramidal layer in naked mole-rats, whereas the hilar region in Cape and highveld mole-rats begins to resemble that of mice and rats (Blackstad, 1956; Amaral, 1978). The continuity of the polymorphic cell layer and CA3 pyramidal cell layer in naked mole-rats is striking, although there is no evidence of a reflected blade of CA3 as seen in primates, humans (Lorente de Nó, 1934; Rosene and Van Hoesen, 1987) and also in some wild rodents (Cavegn et al., 2013).

The prominent CA3 in all mole-rats corresponds well with previous observations that rodents have relatively high numbers of CA3 pyramids (Slomianka et al., 2013). In CA1, lamination into deep, scattered pyramids separated by a cell-sparse zone from a continuous band of superficial cells is very clear in Cape and highveld mole-rats and apparent throughout the septotemporal axis of the hippocampus, further supporting the division of CA1 into sublayers (Slomianka et al., 2011). With the exceptions discussed below, calbindin and parvalbumin staining in the hippocampus of mole-rats resembles that of rats (Sloviter, 1989). Calbindin+ CA3 pyramids in naked mole-rats are distributed similar to CA3 zinc-containing neurons in mice and rats (Slomianka and Geneser, 1997), in which the two markers also colocalize in CA1. Calbindin+ deep CA3 pyramidal cells have been described as a transient feature in fetal rhesus monkeys (Berger and Alvarez, 1996) and fetal humans (Ábrahám et al., 2009). This trait may reflect a partial neoteny, which has also been discussed as a factor in the high anoxia tolerance of naked mole-rats (Larson and Park, 2009). A high anoxia tolerance is also found in embryonic and neonatal mammals [for review see Singer (1999)] supporting the idea that naked mole-rats may show slowed or arrested brain development (Edrey et al., 2011).

## Concluding remarks

We found that high habitat demands are associated with high AHN (Cavegn et al., 2013). AHN is robust but low in all mole-rats reflecting the subterranean live style of the species. In as much as social interactions with conspecifics are part of habitat variability, an increase in sociality should be associated with an increase in AHN. These relations are conserved, not only in mole-rats (Slomianka et al., 2013), even though the wider anatomical context of AHN may differ. Although neoteny may explain some of the cytoarchitectonic characteristics observed in naked mole-rats, it does not extend to the regulation of AHN.

## Author contributions

Irmgard Amrein study design, data analysis, wrote the manuscript, provided funding; Anton S. Becker trapping animals, tissue preparation and processing; Stefanie Engler immunohistochemistry; Shih-hui Huang data acquisition, prepared graphs; Julian Müller data acquisition; Lutz Slomianka data analysis, prepared figures and wrote manuscript; Maria K. Oosthuizen responsible for trapping permits, trapping animals, tissue processing, data acquisition, wrote the manuscript. All authors read and approved the final manuscript.

### Conflict of interest statement

The authors declare that the research was conducted in the absence of any commercial or financial relationships that could be construed as a potential conflict of interest.
